# Michigan State Board of Health

**Published:** 1883-12

**Authors:** 


Secretary.

---

MICHIGAN STATE BOARD OF HEALTH. Reported for the CHICAGO MEDICAL JOURNAL AND EXAMINER.

The regular meeting of the State Board of Health was held October 9, 1883, at its office in the Capitol at Lansing, the following members being present: Arthur Hazlewood, M.D., of Grand Rapids; C. V. Tyler, M.D., of Bay City; J. H. Kellogg, M.D., of Battle Creek; and Henry B. Baker, M.D., Secretary.

The Secretary presented his annual report of property, showing valuable accessions to the Library of the Board by gifts and exchanges; also his quarterly report of work done in the office, the regular correspondence alone, exclusive of many postal card communications not copied, making 572 pages of the letter-book record.

From week to week, as magazines and other accessions to the library are received, considerable work is necessary to make references by which, at any time, papers on a given subject may be found; also to look up papers on various subjects, and to pack and ship books, etc., to those who undertake to write papers for sanitary conventions, or other uses. During the past quarter, three such requests for books and papers have been filled.

A letter from Dr. Whelan, on behalf of the common council of Hillsdale, invited the Board to hold a sanitary convention in that city. The invitation was accepted, the convention to be held at such time as shall be agreed upon hereafter. A letter was read from Rev. J. Pierson, D.D., of Ionia, relating to holding a sanitary convention in that city. It was decided to hold one in Ionia, if practicable, early in December.

A communication from Hon. C. A. Gower, requesting this Board to examine plans for a proposed new building at the State Reform School, was received and acted upon.

A communication was read from Dr. Isler, of the Upper Peninsula, requesting that the documents issued by this Board on the prevention of contagious diseases, be translated and published in the Scandinavian and Finnish languages, for the use of miners and others who do not read English, and among whom both scarlet fever and diphtheria are now present. The leaflet on communicable diseases (No. 47) was ordered to be translated and published in the Scandinavian, French, and Polish languages.

Announcement of the meeting of the American Public Health Association was made, and it was voted to hold a meeting of the Board in Detroit November 18, to attend the meeting of the Association, and to transact such business as may come before the Board.

A communication from the chairman of the Ontario Provincial Board of Health gave notice of a sanitary convention at London, Ontario, November 16 and 17. Drs. Baker and Hazlewood were appointed to attend this convention.

The Secretary was directed to procure from the county clerks, for the uses of the Board, a list of the physicians in the several counties, and their postoffice addresses if practicable.

A memorial was read from the citizens of Morley, Mecosta county, relative to the throwing of dust and other refuse into a stream, a subject not controlled by the State Board, but by the courts. The memorialists had been so informed by the Secretary.

The Secretary presented a *résumé* of the work of other boards of health.

The Boston, Mass., Board of Health has lately placed measles on its list of diseases to be reported to the Board by householders and physicians. That Board has publicly offered to superintend the process of disinfection, if requested so to do by the householder. Dr. Kellogg thought it desirable that boards of health superintend disinfection after contagious diseases, where possible.

He thought disinfection by sulphur would be more efficacious, if carried on in a moist atmosphere.

Drs. Hazlewood and Baker were appointed a committee to examine and report on text-books on hygiene with especial reference to alcohol, if any such books are sent to the Board for examination.

Dr. Kellogg, for himself and Dr. Avery, special committee on the present knowledge respecting diphtheria, reported a paper embodying a large number of replies to a circular of inquiry, some being very valuable. The report was accepted with thanks, and ordered printed in the annual report.

The next meeting of the Board will probably be for the examination of plans for buildings at the State Reform School.

